# Effect of early GnRH antagonist administration on assisted reproductive technique outcomes in normal responders

**DOI:** 10.25122/jml-2021-0286

**Published:** 2022-02

**Authors:** Manal Al-Obaidi

**Affiliations:** 1. Reproductive Physiology, High Institute for Infertility Diagnosis and Assisted Reproductive Technologies, Al-Nahrain University, Baghdad, Iraq

**Keywords:** IVF-ICSI, normal responders, GnRH antagonist sandwich protocol, flexible GnRH antagonist protocol

## Abstract

One of the main questions in assisted reproductive techniques is how to prevent premature LH surge using a variety of protocols depending on either pituitary down-regulation, in GnRH agonist protocols, or by receptors blockage, in GnRH protocols. It is possible to say that GnRH protocols are most popular nowadays. The study aimed to assess the effectiveness of early antagonist administration during days ≤6 and later antagonist administration on days >6 on assisted reproductive outcomes. Women admitted to the ART Department at the High Institute for Infertility Diagnosis and ART, Al-Nahrain University, Baghdad, Iraq were included in the study. Reproductive outcomes were evaluated in early ≤6 and late >6 antagonist administration in a total of 44 normal responders, as follows. Sandwich protocols in 14 patients that received antagonists in the first 3 days of the follicular phase and conventional flexible antagonist protocol in 30 patients. We compared the outcomes between the two groups. There were no differences between early antagonist administration ≤6 and late >6 days in the number of MII oocytes, 2PN, the number of transferred embryos, grades of the embryos, and pregnancy rates. However, there were statistically significant differences between the duration of stimulation and the total Gonadotropin dose required. There was no effect of antagonist administration on days ≤6 and >6 on controlled ovarian stimulation on assisted reproductive outcomes.

## Introduction

The gonadotropin-releasing hormone (GnRH) agonist long protocol played a key role in poor ovarian responders and was used for ovarian stimulation to inhibit the premature surge of luteinizing hormone for *in vitro* fertilization (IVF). Although it had several side effects, this method was widely accepted and used as a long-duration protocol treatment, increasing the pregnancy rate and the number of oocytes retrieved [1]. Different studies and meta-analyses showed major complications leading to higher hospital admission associated with ovarian hyperstimulation syndrome (OHSS) [2]. To overcome these complications, various studies were conducted using GnRH antagonist, which has an immediate mode of action, shorter duration, decreased hospital stay, and it is beneficial to patients undergoing ovarian stimulations [3]. After the introduction of the GnRH antagonist, it was appreciated as additional support for ovarian stimulation in IVF cycles given the patient’s benefits [1]. Several strategies have been suggested to improve the reproductive outcome of the existing GnRH antagonist protocol in the ART setting. These include using the flexible rather than fixed GnRH antagonist regimen [4] and initiation of GnRH antagonist from day 1 of stimulation until the day of hCG administration [5], as well as premenstrual administration of GnRH antagonist [6]. In controlled ovarian hyperstimulation, GnRH antagonist fixed dosing is started on day 5 or 6 of stimulation, and the flexible dosing begins after the follicles reach 13–14 mm in diameter [3–7]. In a prospective randomized study using a lower Gonadotropin dose in the flexible protocol than in the fixed protocol, a higher yield of oocytes was achieved, and no difference was observed between the two protocols concerning the clinical pregnancy rate (CPR) [8]. There is evidence to support a fixed daily injection protocol starting on day 6 or 7 of the menstrual cycle (*i.e.*, 5–6 days after initiation of stimulation) [8]. Gonadotropin-releasing hormone antagonists may be too late when started in the mid-follicular phase in some patients. Several studies have demonstrated that there are hormonal fluctuations during the follicular phase in conventional GnRH antagonist protocol that negatively impact ICSI outcomes. Some studies showed that decreasing high LH levels during the follicular phase may benefit endometrial receptivity and pregnancy rates [9]. Moreover, others have shown no effect on pregnancy rates [10]. Therefore, the aim of this prospective study was to compare the effectiveness of antagonist administration at <6 days and ≥6 days after controlled ovarian hyperstimulation (COH) on assisted reproductive technique (ART) outcomes.

## Material and Methods

This was a prospective comparative study performed at the Higher Institute of Infertility Diagnosis and Assisted Reproductive Techniques, Al-Nahrain University (Baghdad/Iraq), from 2017 to 2019. Forty-four women as normal responders undergoing intracytoplasmic sperm injection cycles were randomized into two groups:

• Inclusion Criteria: normal responder, age group 18–44 years, infertility due to male factors, and couples with unexplained infertility;
• Exclusion Criteria: patients with endocrine disorders, anatomical and pathological abnormalities in the uterus.

### Ovarian Stimulation

Recombinant FSH (rFSH) (Gonal f, Merck Serono Company, Geneva, Switzerland) and the GnRH antagonist Cetrorelix acetate 0.25 mg (Cetrotide, Merck Serono Company, Geneva, Switzerland) were used for controlled ovarian hyperstimulation. The initial Gonadotropin dose was individualized for each patient according to age, body mass index (BMI), antral follicle count, and/or previous responsiveness to ovarian stimulation. Further dose adjustments were performed based on the ovarian response, as evaluated using serum E_2_ measurement and follicular diameter by transvaginal ultrasound, obtained every 2 or 3 days. In the sandwich protocol (n 14), the GnRH antagonist was administered (0.25 mg/d) on days 1, 2, and 3 of the menstrual cycle and stopped after that, to be re-administered when the leading follicle reaches a 13 to 14 mm diameter and continued until hCG day. The patients were administered Gonadotropin beginning on cycle day 3 of ovarian stimulation. In conventional flexible GnRH antagonists (n 30), patients were administered Gonadotropin beginning on CD 2 for ovarian stimulation. By using a flexible protocol, GnRH antagonist injections (0.25 mg) were started as soon as the follicles reached more than 13–14 mm in diameter with a multiple-dose regimen, in which daily antagonist (0.25 mg) was given until the day of hCG injection when two to three leading follicles reached a mean diameter of 18 mm. Human chorionic Gonadotropin (Ovitrelle injections 6500 IU/vial (250 mg) of Human chorionic Gonadotropin (HCG) (Merck-Serono Company, Geneva: Switzerland) was administered when the transvaginal scan showed two or more follicles with a diameter of ≥18 mm (Copperman, and Benadiva 2013). Transvaginal oocyte retrieval was performed 34 to 35 hours after hCG administration under ultrasound guidance. The luteal phase was supported from the day of oocytes retrieval or the day after of oocytes retrieval by vaginal progesterone (Cyclogest® 400 mg twice: Cox Pharmaceuticals, Barnstaple, UK). Serum ß-HCG assay was done on day 14 after the embryo transfer indicative of biochemical pregnancy. A woman with a positive result was later confirmed by an ultrasound examination to objectify the existence of a number of gestational sacs with cardiac activity, indicative of clinical pregnancy. Patients were divided into two groups as follows: early GnRH antagonist administration consisted of patients who reached the criteria for GnRH antagonist administration on stimulation day ≤S6, and late GnRH antagonist administration consisted of patients who started the GnRH antagonist on stimulation day 6 >S6.

### Laboratory Procedures

The handling of oocytes, sperm, zygotes, embryos, and the embryo transfer technique were performed similarly in all women. Briefly, the cumulus oocytes complexes were incubated for 2 hours after retrieval. At this stage, the meiotic status of retrieved oocytes was evaluated after the denudation of its cumulus and corona layers throughout the use of hyaluronidase enzyme and mechanical pipetting. The existence of the first polar body (PBI) designated the gamete as second metaphase (MII) stage oocytes. The intracytoplasmic sperm injection (ICSI) procedure was carried out. Fertilization and pronuclear evaluation were performed 16–18 hours after ICSI (*i.e.*, 50–54 hours after hCG administration). The presence of two pronuclear and two polar bodies characterized normal fertilization. Embryo transfer during the study period was performed 48 or 72 hours after oocytes retrieval. Embryos were scored according to the Istanbul consensus workshop (Alpha Scientist in Reproductive Medicine and ESHRE Special Interest Group of Embryology.2011) and classified into grades 1, 2, 3, in accordance with the classic criteria *(i.e.*, blastomere homogeneity, fragmentation, and the degree of nucleated fragments) [11].

### Statistical analysis

Data were collected, summarized, analyzed, and presented using the statistical package for social sciences (SPSS) version 23 and Microsoft Office Excel 2010. Qualitative (categorical) variables were expressed as number and percentage, whereas quantitative (numeric) variables were first evaluated for normality distribution using the Kolmogorov-Smirnov test, and then normally distributed numeric variables were expressed as mean (an index of central tendency) and standard deviation (an index of dispersion). In contrast, numeric variables that were not normally distributed were expressed as median (an index of central tendency) and interquartile range (an index of dispersion).

## Results

Normal responders exhibited no significant differences in mean age, body mass index, infertility duration, cause of infertility, and type of infertility when categorized according to the type of protocol, sandwich versus conventional antagonist (p>0.05) as in Table 1.

**Table 1.** Comparison of demographic characteristics of normal responders.

**Characteristic**	**Statistic**	**Total (n=44)**	**Sandwich (n=14)**	**Conventional (n=30)**	**P**
**Age (years)**	Mean±SD	28.77±4.90	28.71±3.95	28.80±5.35	0.958 * NS
**BMI (kg/m^2^)**	Mean±SD	28.53±4.41	29.09±4.66	28.27±4.34	0.569 * NS
**Infertility duration (years)**	Median (IQR)	6.50 (6.50)	5.00 (8.00)	7.00 (5.25)	0.276 † NS
**Number of IVF cycles**	Median (IQR)	0.00 (0.00)	0.00 (0.00)	0.00 (0.00)	0.599 † NS
	Range	0.00–2.00	0.00–1.00	0.00–2.00	
**Infertility cause**	Female, n (%)	3 (6.8)	1 (7.1)	2 (6.7)	0.307 ¥ NS
	Male, n (%)	28 (63.6)	10 (71.4)	18 (60.0)	
	Combined, n (%)	1 (2.3)	1 (7.1)	0 (0.0)	
	Unexplained, n (%)	12 (27.3)	2 (14.3)	10 (33.3)	
**Type of infertility**	Primary	27 (61.4)	9 (64.3)	18 (60.0)	0.786 ¥ NS
	Secondary	17 (38.6)	5 (35.7)	12 (40.0)	

n – number of cases; SD – standard deviation; IQR – interquartile range; BMI – body mass index; IVF – in vitro fertilization; * – Independent samples t-test; † – Mann Whitney U test; ¥ – Chi-square test; S – significant at p≤0.05; NS – not significant at p>0.05.

The hormonal status of normal responders concerning the protocol type is shown in Table 2. There was no significant difference in mean serum FSH, LH, and FSH/LH ratio between normal responders undergoing sandwich protocol and those undergoing conventional antagonist protocol (p>0.05). There were no significant differences in the mean serum E_2_, prolactin, and TSH levels between sandwich and conventional antagonist groups in normal responders (p>0.05), as shown in Table 2.

**Table 2.** Comparison of hormonal status in normal responders between sandwich and conventional protocols.

**Hormone**	**Statistic**	**Total (n=44)**	**Sandwich (n=14)**	**Conventional (n=30)**	P *
**FSH (mIU/ml)**	Mean±SD	6.52±3.07	5.90±2.88	6.81±3.16	0.368 NS
**LH (mIU/ml)**	Mean±SD	3.79±1.65	4.06±1.77	3.66±1.61	0.456 NS
**FSH/LH**	Mean±SD	1.92±1.00	1.54±0.63	2.09±1.09	0.088 NS
**E_2_ (pg/ml)**	Mean±SD	29.41±14.79	24.64±6.72	31.64±16.96	0.145 NS
**Prolactin (ng/ml)**	Mean±SD	16.81±11.70	16.68±14.89	16.87±10.19	0.960 NS
**TSH (mIU/ml)**	Mean±SD	1.72±0.53	1.77±0.61	1.70±0.50	0.658 NS

n – number of cases; SD – standard deviation; FSH – follicle-stimulating hormone; LH – luteinizing hormone; E_2_ – estradiol; TSH – thyroid-stimulating hormone; * – Independent samples t-test; significant at p≤0.05; NS – not significant at p≥0.05.

Women in late GnRH administration required a significantly longer ovarian stimulation period and significantly higher dose of Gonadotropin, as shown in Table 3. There were no differences between early and late GnRH administration regarding total oocytes, the number of MII oocytes, the number of 2PN, the fertilization rates, and the number of transferred embryos or grade of the embryos, as shown in Table 3.

**Table 3.** Response to ovarian stimulation in the early ≤S6 and late >S6 GnRH antagonist administration in normal responders group.

Characteristics	Early (≤day S6)		Late (>day S6)		P
	Mean	SD	Mean	SD	
**Stimulation days**	6.71	0.49	9.41	1.21	<0.001
**Gonadotropin**	12.71	1.25	21.81	7.50	0.003
**E_2_ at trigger**	1192.80	487.75	1477.10	790.67	0.366
**Progesterone at trigger day**	0.32	0.26	0.80	0.60	0.199
**Progesterone at ova pickup**	4.04	2.10	6.11	3.99	0.191
**Total oocytes**	11.57	5.88	9.73	4.86	0.379
**MII**	8.43	5.09	6.03	3.69	0.145
**2 PN oocytes**	5.29	3.45	4.81	2.75	0.689
**G1percent**	40.47	21.22	43.10	29.18	0.822
**G2percent**	43.66	24.32	50.78	28.76	0.543
**G3percent**	1.59	4.20	6.12	15.55	0.452
**Number of embryo transfer**	3.00	1.41	2.95	1.20	0.916

SD – standard deviation; E_2_ – estradiol; MII oocytes – mature oocytes; PN – pronuclear; G – grade.

In both sandwich and conventional antagonist protocol, women in late GnRH administration required a significantly longer ovarian stimulation period and a significantly higher dose of Gonadotropin, as shown in Table 4. There were no differences between early and late GnRH administration regarding total oocytes, number of MII oocytes, fertilization rates, number of transferred embryos or grade of the embryos except for the number of 2PN, which was significantly higher in sandwich protocol than conventional antagonist protocol in early and late GnRH administration, as shown in Table 4.

**Table 4.** Response to ovarian stimulation in the early ≤S6 and late >S6 GnRH antagonist administration in normal responders group according to protocols.

Characteristic	Sandwich early ≤S6		Conventional early ≤S6		Sandwich late>S6		Conventional late>S6		P
	Mean	SD	Mean	SD	Mean	SD	Mean	SD	
**Stimulation days**	6.50 B	0.58	7.00 B	0.00	9.00 A	0.94	9.56 A	1.28	<0.001
**Gonadotropin**	12.25 B	1.26	13.33 B	1.15	22.80 A	5.81	21.44 A	8.10	0.029
**E_2_ at trigger**	1157.80	518.25	1239.60	552.36	1635.40	643.58	1418.0	842.03	0.705
**Progesterone at trigger day**	0.32	0.26	.	.	1.02	0.92	0.71	0.41	0.274
**Progesterone at ova pickup**	3.40	0.85	4.90	3.19	5.62	3.01	6.32	4.37	0.540
**Total eggs**	13.75	4.57	8.67	7.09	12.70	6.41	8.63	3.72	0.054
**MII**	9.50	4.43	7.00	6.56	7.90	4.82	5.33	3.00	0.115
**2 PN oocytes**	7.50 A	2.38	2.33 C	2.08	6.60 A	3.78	4.15 B	1.96	0.006
**G1percent**	50.00	13.64	27.77	25.46	47.91	28.82	41.32	29.65	0.685
**G2percent**	47.23	18.45	38.90	34.71	43.67	19.49	53.41	31.42	0.715
**G3percent**	2.78	5.55	0.00	0.00	8.43	16.31	5.27	15.49	0.813
**Number of embryo transfer**	3.50	0.58	2.33	2.08	3.10	1.20	2.89	1.22	0.630
**Biochemical pregnancy rate**	2/4		0/3		7/10		12/27		0.183
**Clinical pregnancy rate**	2/4		0/3		7/10		12/27		0.183
**OHSS**	0/4		0/3		1/10		4/27		0.745

There was no significant difference in pregnancy rate between early and late GnRH administration in total normal responders and sandwich and conventional antagonist protocol (Table 5).

**Table 5.** Pregnancy outcome according to antagonist starting day.

**Group**	**Method**	**GnRH-antagonist start day**	**Total**	**Biochemical pregnancy Positive**		**P**
				**n**	**%**	
**Normal total**	**Con.ant.+sand**	Early ≤6	10	3	30.0	0.202 NS
		**Late >6**	34	18	52.9	
**Normal**	Conventional	**Early ≤6**	9	3	33.3	0.626 NS
		**Late >6**	21	9	42.9	
		**Total**	30	12	40.0	
	**Sandwich**	**Early ≤6**	1	0	0.0	0.164 NS
		**Late >6**	13	9	69.2	
		**Total**	14	9	64.3	

## Discussion

This study was dependent on the effect of starting the day with GnRH antagonist administration in a multiple-dose protocol (0.25 mg/day), in stimulation days ≤6 and >6 of controlled ovarian stimulation on assisted reproductive technique (ART) outcomes. No differences were found between the outcome parameters of the ICSI cycle. The 2 PN oocytes were not significantly different between patients receiving GnRH antagonist administration before or after stimulation day 6, although significantly higher in patients undergoing sandwich protocol in patients receiving GnRH antagonist administration before or after stimulation day 6. This agrees with Blockeel *et al.*, who showed a higher number of 2PN oocytes in early short follicular antagonist protocol (sandwich protocol) [12]. Using sandwich protocol in another study showed improved maturation and fertilization rates of the oocytes [13]. The explanation of these results could be attributed to the modification of the flexible GnRH antagonist regimen (sandwich protocol) consisting of GnRH antagonist supplementation on days 1, 2, and 3 of the same cycle. This modification will improve the number of mature oocytes and the efficiency of the oocytes, improving their normal fertilization rate. This has been achieved by significantly lowering early follicular serum FSH and LH levels [13]. While a possible detrimental effect of GnRH antagonist on the oocytes and embryo was shown in the past, studies do not recognize such an effect. High-dose Gonadotropin in the ICSI cycles may have a negative impact on oocytes, embryo quality, and endometrial receptivity [14]. One study showed that GnRH antagonist administration after day 6 of stimulation has a detrimental effect on pregnancy outcomes [7]. However, multiple studies have shown that starting GnRH antagonist either before stimulation day 6 or after stimulation day 6 does not affect pregnancy outcomes [15]. There was no difference between the GnRH antagonist administration before or after stimulation day 6 concerning pregnancy outcomes in the current study. The aim of GnRH antagonist administration during *in vitro* fertilization (IVF) cycles is to prevent premature luteinization and LH surge [16]. Therefore, if there is no progesterone increase on hCG day, it is expected that there will be no difference in pregnancy rates between GnRH antagonist administration either before stimulation day 6 or after stimulation day 6 in the groups. The progesterone levels were low (<1.5 ng/mL) in both groups in the present study [17].

## Conclusion

There was no effect of antagonist administration on days ≤6 and >6 of controlled ovarian stimulation on assisted reproductive outcomes. However, there were statistically significant shorter duration of stimulation and less total gonadotropin dose required in antagonist administration on days ≤6.

## Acknowledgments

### Conflict of interest

The author has declared no conflict of interest.

### Ethical approval

The study was approved by the Local Medical Ethical Committee of the High Institute for Infertility Diagnosis and Assisted Reproductive Technologies, Al-Nahrain University (849/06/2017).

### Consent to participate 

Written informed consent was obtained from the participants.

### Data availability

Further data is available from the corresponding author on reasonable request.

### Personal thanks

The author is grateful for the patient’s cooperation.

### Authorship

All parts of the manuscript were done by MAO.
